# Nicotine delivery from the refill liquid to the aerosol *via* high-power e-cigarette device

**DOI:** 10.1038/s41598-017-03008-0

**Published:** 2017-06-01

**Authors:** Nathalie Prévôt, Fabien de Oliveira, Sophie Perinel-Ragey, Thierry Basset, Jean-Michel Vergnon, Jérémie Pourchez

**Affiliations:** 1 INSERM, U1059, F-42023 Saint Etienne, France; 2 Université de Lyon, F-69000 Lyon, France; 3 CHU Saint-Etienne, Department of Nuclear Medicine, Saint-Etienne, F-42055 France; 4 Ecole Nationale Supérieure des Mines de Saint-Etienne, CIS-EMSE, SAINBIOSE, F-42023 Saint Etienne, France; 5 CHU Saint-Etienne, Medical-Surgical Intensive Care Unit, Saint-Etienne, F-42055 France; 6 CHU de Saint-Etienne, Laboratoire de Pharmacologie–Toxicologie, F-42055 Saint Etienne, France; 7 CHU Saint-Etienne, Department of Chest Diseases and Thoracic Oncology, Saint-Etienne, F-42055 France

## Abstract

To offer an enhanced and well-controlled nicotine delivery from the refill liquid to the aerosol is a key point to adequately satisfy nicotine cravings using electronic nicotine delivery systems (ENDS). A recent high-power ENDS, exhibiting higher aerosol nicotine delivery than older technologies, was used. The particle size distribution was measured using a cascade impactor. The effects of the refill liquid composition on the nicotine content of each size-fraction in the submicron range were investigated. Nicotine was quantified by liquid chromatography coupled with tandem mass spectrometry. Particle size distribution of the airborne refill liquid and the aerosol nicotine demonstrated that the nicotine is equally distributed in droplets regardless of their size. Results also proved that the nicotine concentration in aerosol was significantly lower compared to un-puffed refill liquid. A part of the nicotine may be left in the ENDS upon depletion, and consequently a portion of the nicotine may not be transferred to the user. Thus, new generation high-power ENDS associated with propylene glycol/vegetable glycerin (PG/VG) based solvent were very efficient to generate carrier-droplets containing nicotine molecules with a constant concentration. Findings highlighted that a portion of the nicotine in the refill liquid may not be transferred to the user.

## Introduction

Nicotine is an addictive psycho-active drug. Smokers are bio-behaviorally addicted, and this is why smoking cessation is very difficult. Medications to stop smoking and nicotine replacement therapy (NRT, *e.g*. adhesive patches, gums, throat lozenges, nasal or mouth sprays, etc.) exist since several decades. NRT generally focuses on the neuropharmacology of nicotine. Thus it fails to address the bio-behavioral component^1^, even if the use of smoking cessation medications and NRT products allows to double quit rates^2^. Electronic cigarettes are battery-powered aerosol devices, which can deliver airborne particles containing nicotine. Electronic nicotine delivery systems (ENDS) appear as a more accurate and appropriate denomination for these aerosol devices rather than electronic cigarettes. Users (*i.e*. “vapers”) inhale doses of vaporized nicotine from a handheld aerosol device. Contrary to smoking tobacco cigarette, ENDS deliver nicotine-containing aerosol (*i.e*. “vape”) without any combustion or smoke. ENDS clearly appear as a disruptive technology that revolutionizes NRT since these devices address for the first time the biochemical and behavioral aspects of smoking addiction. After several years of technological development, since the ENDS invention in 2003, the ENDS business increased dramatically around 2010. Nowadays ENDS have surpassed sales of usual NRT despite its 35 years on the market. Against this background, some analysts forecast that ENDS will surpass the sales of smoking tobacco cigarettes within 10 years^3^.

Despite a steep increase in market penetration, very few clinicians and researchers are committed in the ENDS scientific research while many issues remain, mainly about safety assessment. Even if it is now quite well-accepted in the scientific community that ENDS provide a harm reduction for smokers who want to quit smoking cigarette^4^, the public health benefit at the population level is actively debated worldwide. ENDS present important regulatory challenges to policy makers and governments. Toxicants resulting from heated flavorings, irritant humectants (mainly glycerol and propylene glycol) or aldehydes emitted by thermal decomposition of ENDS liquid components can induce adverse effects on the respiratory system^5^. But the assessment of long-term effects of ENDS is complicated due to scientific uncertainties, such as the knowledge of chemicals doses inhaled by vapers, but also the control of the aerosol nicotine delivery.

In perfect accordance with the global objective of smoking reduction or cessation, the development of more effective ENDS technologies seems desirable. The main purpose is to offer an increased and well-controlled nicotine delivery first to the aerosol, and then to the vaper. Recently, new technologies are developed thanks to various technological breakthroughs. In this frame, high-power ENDS can deliver high levels of aerosol nicotine^6^. Indeed, at high voltage, this new generation of ENDS appears to generate aerosol nicotine in more consistent quantities (2.72–10.61 mg of nicotine/20 puffs) than older ENDS design based on cartomizers technology (1.01–3.01 mg of nicotine/20 puffs), but also than smoking tobacco cigarettes (1.76–2.20 mg of nicotine/cigarette). Consequently, high-power ENDS seem to be a very promising technology to enhance the aerosol nicotine delivery. This study deals with nicotine delivery to the aerosol by recent high-power ENDS. The aerosol output and the aerodynamic particle size distribution were measured using inertial sizing techniques, (*i.e*. cascade impaction). This work proposes for the first time an exhaustive characterization of the airborne nicotine flux including the nicotine concentration assessment for aerodynamic particle fractions from 7 nm to 10 µm.

## Methods

### Materials

A recent high-power ENDS was used (purchased in March 2016 from a local store and online distributor). This ENDS model was made up of a variable lithium-ion battery (iStick TC40 W, Eleaf) and an atomizer (GS Tank, Eleaf). Equipped with an internal 2600 mAh battery, the variable wattage can be adjusted up to 40 watts of vaping power. Using pure nickel or titanium coils, a complete control over the temperature of the atomizer coil is possible. The temperature control resistance range is 0.05–1 ohm and the variable wattage/voltage resistance range is 0.15–3.55 ohms. The GS-Tank is a recently engineered atomizer, requiring maximum push power ranging from 20 W to 40 W. The nickel heating wire and the head with pure cotton wick used in the atomizer makes it compatible with the temperature controlled batteries. This resistance is 0.15 ohm. Prior to performing particle size experiments, batteries were fully charged, the maximum air inflow position was fixed, and the value of the electrical resistance was checked. Atomizers were changed regularly during the experimental campaign to avoid biases due to the use of degraded and/or dirty coil. In our study, all combinations of vaping parameters (specifically at high voltage) were carefully adjusted to avoid the dry puff phenomenon. The human control feedback by a regular vaper was used to be certain of the absence of unpleasant taste using the ENDS. For all experiments the power level of the battery was fixed at 15 W.

Two different compositions of refill liquid were used corresponding to the 80% PG + 20% VG (noted 80PG/20VG) and 20% PG + 80% VG (noted 20PG/80VG); PG referred to propylene glycol, and VG referred to vegetable glycerin. These formulations were home-made from commercial solutions with a nicotine concentration of 18 mg.L^−1^ (purchased in March 2016 from a local store, 100-VG and 100-PG base, A&L company, France). It is important to underline that both formulations of refill liquid used for this study were flavors-free in order to study only the impact of the ratio PG/VG on aerosol nicotine delivery. Although the nominal capacity of the tank-type atomizer was 3 mL, it was filled with 2 mL of the prepared solution to avoid potential overfilling.

### Particle size distribution

Aerosol particle sizing was defined in terms of Mass Median Aerodynamic Diameter (MMAD). Aerodynamic particle size distribution was measured using inertial sizing techniques, and more precisely cascade impaction which is the method specified for regulatory approval of inhalation product in the pharmaceutical sector^7^. It is the only sizing technique which presents the capability to differentiate different components in the mainstream of ENDS aerosol: humectants (*i.e*. VG and PG) and the nicotine (*i.e*. the active pharmaceutical ingredient). Indeed, multistage cascade impactors are used to size-fractionate a sample, uniquely enabling measurement of the particle-size distribution of the active ingredient, rather than of the complete formulation. The resulting information is crucial when assessing the likely deposition behavior of the drug during inhalation. As a result, the instrument of choice for measuring the aerodynamic size distribution of inhaled products for both regulators and pharmacopoeias alike is the cascade impactor.

**Figure 1:**
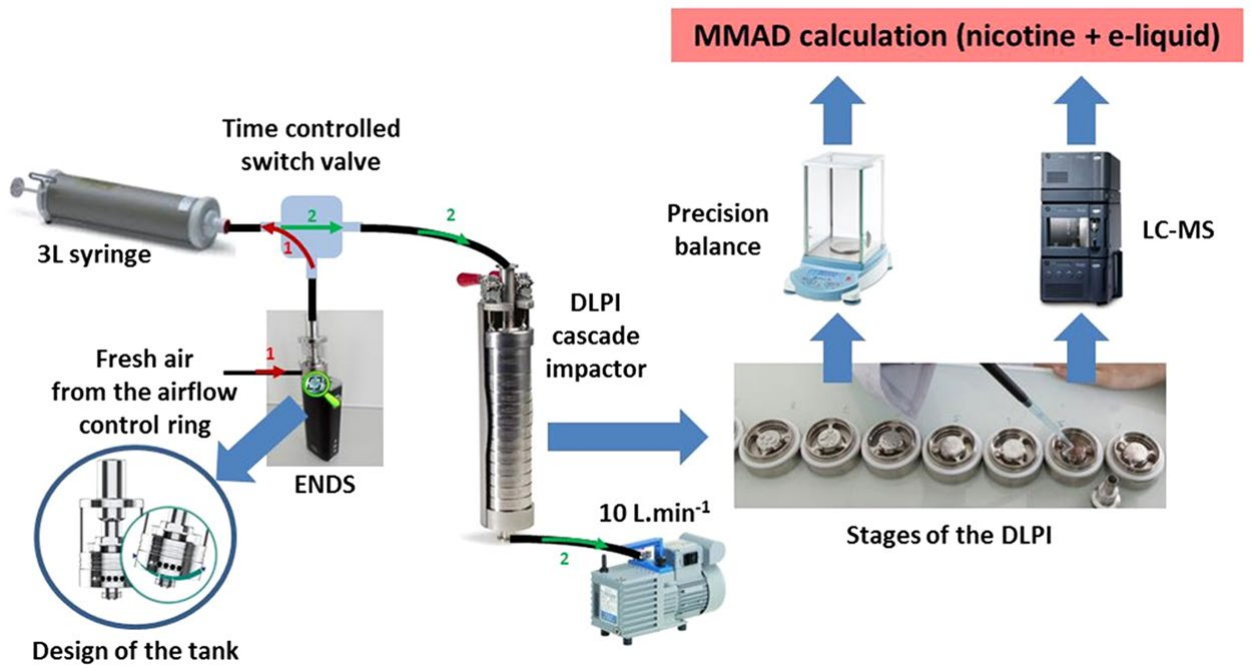
Scheme of the experimental set-up for the particle sizing distribution of aerosol generated by ENDS.

Special care was taken to develop a measurement strategy in order to limit experimental bias due to particles evaporation (due to high dilution ratios) or particles coagulation (due to long residence times between aerosol sampling and measurement). The Dekati Low-Pressure Impactor (DLPI) and the Electrical Low-Pressure Impactor (ELPI) allows the collection of atomized particles from 7 nm to 10 µm into 12 size fractions and operates with an air flow of 10 L.min^−1^. It consists of a 12-stage cascade low pressure impactor leading to determine gravimetric size distribution using an electronic precision balance (Adventurer Pro, OHAUS, USA). An in-house interface was designed to introduce quickly and reproducibly a well-controlled volume and duration puff into the inlet of the impactor. This interface was composed of a 3 L syringe (Hans-Rudolph, USA) connected to both ENDS and DLPI cascade impactor (Fig. 1). Aerosol sampling was carried out considering 4-s puff (with a flow rate of 500 mL.s^−1^) and a dilution ratio of 1.5 (2L of aerosol diluted in 1L of ambient air initially present in the 3L-syringe). The syringe acts as a reservoir during all the puffing process duration. At the end of the 4-s puff duration, the volume of aerosol contained in the syringe was injected into the DLPI set-up using the 10 L.min^−1^ flowrate of the cascade impactor without any other air dilution to limit the evaporation process of the volatile airborne droplets. However, the main drawback of this protocol is to have disparate flowrate (500 mL.s^−1^) compared to flowrate (10–50 mL.s^−1^) used by vapers in real-life practice. Nevertheless, we must keep in mind that we used a third ENDS generation equipped with an airflow control ring. The amount of airflow can easily be adjusted by the control ring on the atomizer base. Our experiments were performed using the maximum air inflow position. Besides using a high flowrate of puffing allows the great advantage to obtain an important volume of aerosol (to avoid high dilution during the aerosol sampling and then the size-fractionation through the DLPI set-up, and thus an evaporation phenomenon which will change the particle size) with a short duration (because of the half*-*life of the aerosol generated by ENDS is limited at approximately 15 seconds).

### Nicotine concentration assessment

After each DLPI measurements, each stage, corresponding to a well-defined aerodynamic size-fraction was rinsed with 1 mL of deionized water into volumetric flasks. Liquids were then assayed for nicotine concentration by liquid chromatography coupled with tandem mass spectrometry (LC-MS). Internal standards [13C, 2H3]-nicotine (Alsachim, France) were used to prepare primary stock solutions stored at −20 °C. Working internal standard solutions were prepared freshly on each day of analysis as serial dilutions in water. Nicotine samples were stored at −20 °C until analysis. Chromatographic analysis was performed using an Acquity ultra-performance liquid chromatograph system using an UPLC HSS-T3 C18 column (Waters, France). The column temperature was maintained at 40 °C. The mobile phase was a mix of A: distilled water containing 0.1% formic acid and B: acetonitrile containing 0.1% formic acid. The flow rate was 0.5 mL.min^−1^. The liquid chromatograph system was coupled to a Xevo TQS Micro triple quadrupole mass spectrometer (Waters, France). The system control and data acquisition were performed using MassLynx V4.1 software (Waters, France).

## Results

### Impact of PG/VG ratio on MMAD

**Figure 2:**
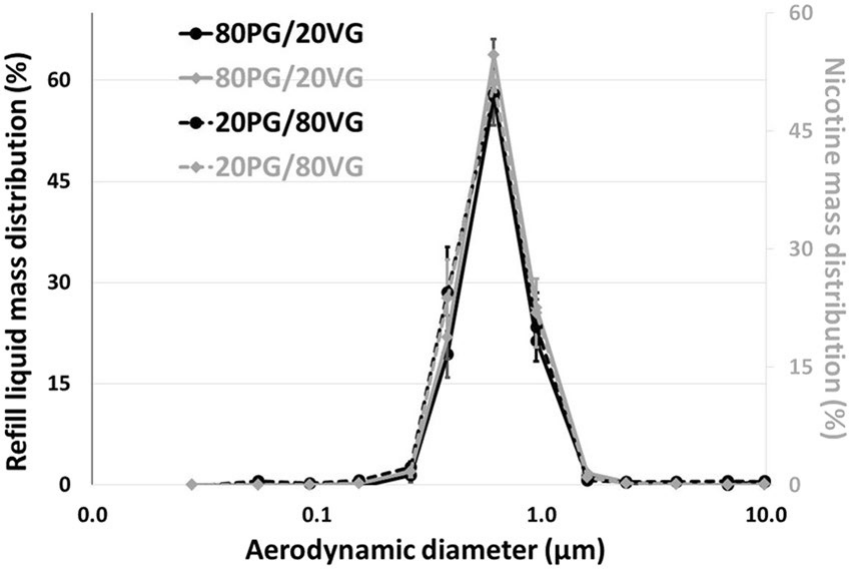
DLPI Impactor-collected data showing the impact of the PG/VG ratio on the frequency mass distribution. Mass of nicotine is in grey (expressed in %) mass of refill liquid in black (expressed in %). The 80PG/20VG formulation was plotted with continuous lines. The 20PG/80VG formulation was plotted with dotted lines. Experiments were performed in triplicate with a power level fixed at 15 W, a dilution ratio of 1.5 and a same volume of aerosol collected into the cascade impactor for all experimental conditions.

Figure 2 shows the frequency mass distribution of both airborne refill liquid and nicotine *vs*. aerodynamic diameter. Table 1 summarizes size data (mainly the MMAD) obtained for all experimental conditions. The PG/VG ratio had clearly no impact on the frequency mass distributions, regardless of the mass of refill liquid (the airborne carrier) or the mass of nicotine (the active product inside this carrier). Besides, refill liquid MMAD (0.76 ± 0.03 µm *vs*. 0.79 ± 0.01 µm), were not statistically different between the 20PG/80VG and 80PG/20VG formulation (Student’s t-distribution with p = 0.30, Table 1). Similar results were observed for Nicotine MMAD (0.77 ± 0.03 µm *vs*. 0.79 ± 0.01 µm; Student’s t-distribution with p = 0.57, Table 1).

**Table 1:** Summary of the size distribution data.

	Aerosol mass distribution		Aerosol nicotine delivery				
	MMAD (µm)	GSD	MMAD (µm)	GSD	Mass of nicotine (mg)	Un-puffed nicotine concentration (mg/mL)	Aerosol nicotine concentration (mg/mL)
20PG/80VG	0.76 ± 0.03	1.49 ± 0.03	0.77 ± 0.03	1.46 ± 0.06	1.42 ± 0.12	21.6 ± 0.9	13.4 ± 2.1
80PG/20VG	0.79 ± 0.01	1.43 ± 0.04	0.79 ± 0.01	1.47 ± 0.02	2.06 ± 0.12	21.1 ± 0.7	16.0 ± 0.7

Experiments were performed in triplicate with a power level fixed at 15 W, a dilution ratio of 1.5 and a same volume of aerosol collected into the cascade impactor for all experimental conditions. MMAD referred to the Mass Median Aerodynamic Diameter. GSD referred to the geometric standard deviation of the mass aerosol distribution.

### Impact of PG/VG ratio on the nicotine delivery

**Figure 3:**
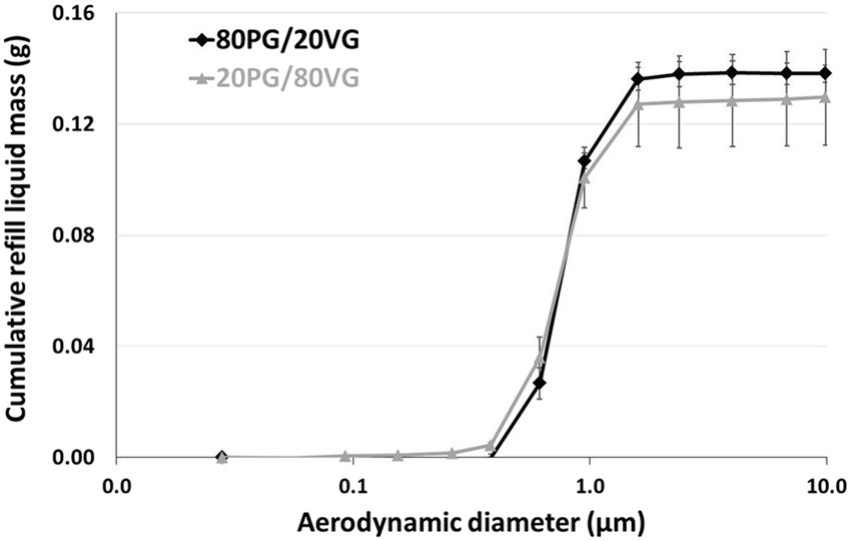
DLPI Impactor-collected data showing the impact of the PG/VG ratio on the cumulative mass distribution of refill liquid (expressed in mg). Experiments were performed in triplicate with a power level fixed at 15 W, a dilution ratio of 1.5 and a same volume of aerosol collected into the cascade impactor for all experimental conditions.

**Figure 4:**
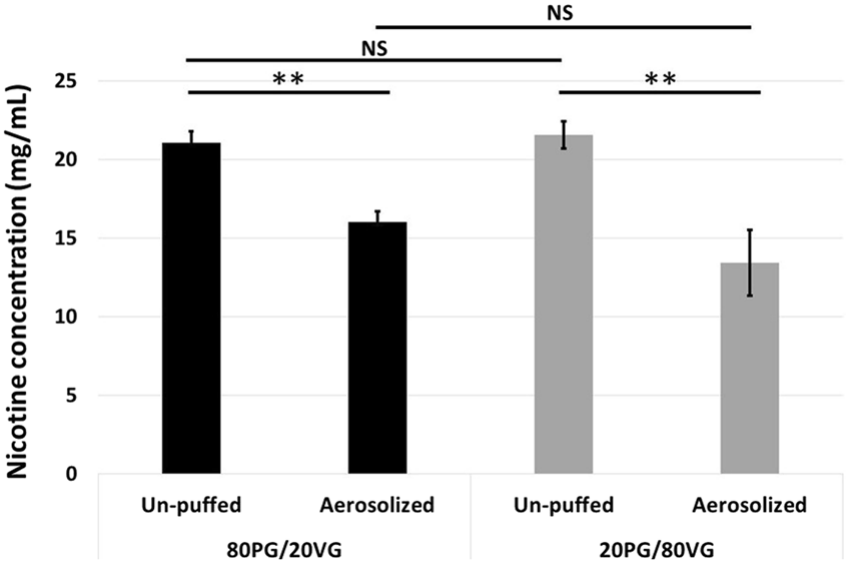
Impact of the PG/VG ratio on the un-puffed and aerosolized nicotine concentration (expressed in mg per mL of refill liquid solution). Experiments were performed in triplicate with a power level fixed at 15 W, a dilution ratio of 1.5 and a same volume of aerosol collected into the cascade impactor for all experimental conditions.

First of all, Fig. 2 perfectly demonstrates that, whatever the PG/VG ratio, a perfect matching between the frequency mass distribution of the airborne refill liquid and the nicotine delivery was observed. Moreover, MMAD of nicotine distribution *vs*. MMAD of refill liquid were similar for the 20PG/80VG (0.76 ± 0.03 µm *vs*. 0.77 ± 0.03 µm, not statistically different regarding to Student’s t-distribution with p = 0.76, Table 1) and the 80PG/20VG formulation (0.79 ± 0.01 µm *vs*. 0.79 ± 0.01 µm, not statistically different regarding to Student’s t-distribution with p = 1, Table 1). Even if no impact of the PG/VG ratio was noticed on the nicotine frequency mass for each aerosol size fraction, a moderate effect of the PG/VG ratio was observed on the nicotine total mass delivery (Table 1). The aerosol nicotine delivery seems to be slightly higher when the PG content of the refill liquid formulation is high (1.42 ± 0.12 mg *vs*. 2.06 ± 0.12 mg, statistically different regarding to Student’s t-distribution with p = 0.003, Table 1). However, Fig. 3 highlights the cumulative mass distribution of the refill liquid. Regarding to the standard deviations, similar mass of refill liquid was aerosolized whatever the PG/VG ratio. Finally, the aerosol nicotine concentration was calculated thanks to these previous data (the aerosol nicotine mass and the aerosol refill liquid mass). Figure 4 shows that the aerosol nicotine concentration is significantly different when comparing aerosol nicotine *vs*. un-puffed refill liquid, with a decrease of 24 ± 7% for the 80PG/20VG formulation (21.1 ± 0.7 mg/mL *vs*. 16.0 ± 0.7 mg/mL, Table 1 and Fig. 4) and a decrease of 38 ± 15% for the 20PG/80VG formulation (21.6 ± 0.9 mg/mL *vs*. 13.4 ± 2.1 mg/mL, Table 1 and Fig. 4). The aerosol nicotine concentration appeared to be similar whatever the PG/VG ratio (13.4 ± 2.1 mg/mL *vs*. 16.0 ± 0.7 mg/mL, not statistically different regarding to Student’s t-distribution with p = 0,17, Table 1 and Fig. 4). Moreover, the initial concentration of nicotine in the refill liquid was slightly different from the value indicated by the manufacturer (21.6 ± 0.9 mg/mL and 21.1 ± 0.7 mg/mL *vs*. 18 mg/mL for the labeled nicotine concentration).

## Discussion

This study deals with the aerosol nicotine delivery by recent high-power ENDS. To the best of our knowledge, this paper proposed for the first time an exhaustive characterization of the airborne nicotine flux including the nicotine content for aerodynamic particle size-fractions from 7 nm to 10 µm. Results perfectly demonstrated that the PG/VG ratio has no significant effect on the frequency distribution of the mass of refill liquid (the airborne carrier) as well as the mass of nicotine (the active product inside this carrier). Whatever the PG/VG ratio, a perfect matching between the frequency mass distribution of the airborne refill liquid and the nicotine was observed. This finding leads to indicate that:

(i) There is no empty airborne carrier generated by recent high-power ENDS (*i.e*. droplet of refill liquid without nicotine).
(ii) The nicotine concentration inside droplets of refill liquid, whatever the aerodynamic size-fractions in the submicron range, is constant for a given PG/VG ratio of refill liquid.
(iii) The particle size distribution of the airborne refill liquid perfectly fits the particle size distribution of the aerosol nicotine.

Besides, this last point, *i.e*. the fact that nicotine is equally distributed in droplets regardless of their size, can appear quite obvious. Indeed, the studied system is a homogenous solution of miscible liquids. Consequently, each droplet contains the same proportion of each components (PG, VG and nicotine). Aerosol droplets are formed after condensation of mixed vapor produced by heating of the refill liquid in the ENDS device. This process may change the composition of the condensate comparing to the un-puffed solution (by the principle known from distillation processes, *e.g*. evaporation and subsequent condensation of alcohol solutions). Nevertheless, the whole condensed liquid has the same composition in term of proportion of PG, VG and nicotine. However, volatile aerosol droplets, and especially in the submicron-sized range such as particles produced by recent high-power ENDS, can lose solvents by evaporation prior to entry into the user’s mouth, or an inertial sizing device such as cascade impactors. As a result, evaporation can cause an increase of nicotine concentration in the droplet and reduction in size. The impact of the evaporation phenomena can be particularly great, for smaller airborne particles compared to larger ones (droplet curvatures have also a strong effect on the evaporation process), when a large volume of dilution air is mixed with the aerosol stream during the sampling step. Nicotine was equally distributed in droplets regardless of their size and the aerosol concentration of nicotine appeared lower than the un-puffed solution. Although the use of a high flowrate for aerosol generation as well as a dilution ratio of 1.5, this finding doubtless proved that no important evaporation bias occurs during the sizing measurement strategy including high flowrate (500 mL.s^−1^, DLPI set-up, sampling including an initial dilution ration of 1.5).

Few studies were devoted to aerosol features generated by ENDS in term of MMAD measurement. The findings obtained in this study (in the 0.75–0.8 µm range of MMAD) appear in good accordance with cascade impactor data previously published^8–11^. Indeed, the results found in the literature, using various types and brands of ENDS, are often in the 0.5–1 µm range of MMAD when inertial sizing techniques were used: 487 nm to 631 nm for Alderman *et al*.^8^, from 600 to 650 nm for Bertholon *et al*.^9^, from 600 to 800 nm for Kane *et al*.^10^ and 1.03 µm for Lerner *et al*.^11^). The use of an important flowrate (500 mL.s^−1^) compared to flowrate (around 20 mL.s^−1^) used by vapers in real-life practice could have been a drawback for this work. It is possible that high flow rate can affect the particle size distribution. For example, the wick may not have enough time to be saturated with liquid and the residence time should be reduced. Besides, we must keep in mind that we used for this work a newly engineered atomizer including an airflow control ring. As previously showed, no important evaporation bias occurs during the sizing measurement strategy and thus the important flowrate used seems do not strongly affect the MMAD determined.

To more precisely evaluate the bias induced by the high flow rate used, experiments were also performed using the ELPI mode of the DLPI cascade impactors with a puffing behavior similar to real-life practices. Within ELPI, particles are electrically charged via a corona charger and subsequently impacted in one of the 12 size fractions stages of the DLPI impactor. The induced current of impacted particles is measured and related to the aerodynamic cut-off diameter of the impactor stage, providing real-time size-dependent distributions. Results can be expressed by means of number concentrations to calculate the Count Median Aerodynamic Diameter (CMAD). Aerosol sampling was carried out considering 4-s puff and a volume of 60 mL (flow rate of 15 mL.s^−1^). An in-house interface was designed. Puffs were performed using a 60 mL syringe connected to the ENDS (exactly as described in Fig. 1, replacing the 3L-syringe with a 60mL-syringe). In these experimental conditions, results showed a CMAD of 0.72 ± 0.01 µm (GSD of 2.21) for the 80PG/20VG formulation. We can convert CMAD into MMAD since for a log-normal distribution the known relationship holds that MMAD = CMAD exp[3(log(GSD))^2^]. Consequently, using this equation we found a MMAD equal to 1.028 µm (conditions: 4-s puff, 60 mL of aerosol, flowrate of 15 mL.s^−1^, no dilution during the sampling using the 60mL-syringe, CMAD calculation using ELPI and then calculation of MMAD) versus 0.79 µm (Table 1, conditions: 4-s puff, 2 L of aerosol, flowrate of 500 mL.s^−1^, dilution ratio of 1.5 during the sampling using the 3L-syringe, DLPI size-fractionation and mass measurement using a precision balance). Many parameters vary between these two experimental conditions (and not only the flow rate). As a result, we assume that the possible bias using a high flow rate, if it exists, induces an error of at most 20% on the measurement of the MMAD. All things considered, this study demonstrated that our data on aerosol sizing are quite relevant compared to the literature on MMAD determination.

Another important finding of this study is to highlight a lower aerosol concentration of nicotine compared to un-puffed nicotine concentration of the refill liquid initially introduced inside the tank-type atomizer. A decrease ranging from 24 ± 7% to 38 ± 15% was measured depending on the PG/VG ratio of the refill liquid. No experimental biases could explain these results (*i.e*. all possible biases, mainly evaporation process of volatile droplets, would have tended to increase aerosol nicotine concentration, not to decrease it). Furthermore, our findings appear highly coherent with results recently published^12^. The consistency of our results with the literature data, again confirm that our findings are relevant although the high flow rate used (which seems to play a minor role when the airflow control ring is at the maximum position). Pagano *et al*. studied the portion of nicotine delivered *vi*a aerosolization using ENDS technology dating from early 2014. They demonstrated that, under their experimental conditions and for their given ENDS technology, the portion of aerosol nicotine delivered to filter pads was often less than half that which was available, indicating that most of the nicotine may be left in the ENDS upon depletion. Several assumptions could be hypothesized to explain this phenomenon:

(i) A partial thermal degradation of the nicotine molecules.
(ii) A part of the nicotine can be present in the gaseous state (*i.e*. in a vapor rather than contained in airborne liquid droplets), so it cannot be collected in the cascade impactor.
(iii) The ENDS process can change the composition of the condensate comparing to the un-puffed solution (the so-called “distillation process”).

Besides, the initial concentration of nicotine in the refill liquid (*i.e*. the un-puffed liquid) was slightly different from the value indicated by the manufacturer (21.6 ± 0.9 mg/mL and 21.1 ± 0.7 mg/mL *vs*. 18 mg/mL for the labeled nicotine concentration). Our analyses of un-puffed liquid by LC-MS highlighted that the nicotine content of refill liquid can be considerably different from manufacture’s labeling. There have been relatively few studies on the accuracy of the labeled nicotine concentrations on refill liquid for ENDS^13–15^. Our findings are perfectly in accordance with a previous work highlighting measured nicotine concentrations higher than the labeled values, with many being over 20% higher^16^.

When delivered through the pulmonary route (as with traditional smoking cigarette or with ENDS), nicotine from the aerosol can theoretically be rapidly absorbed into the blood circulation. Crossing the alveolar*-*capillary barrier, aerosol nicotine reaches the brain within few seconds^17^. By contrast, buccal and dermal nicotine absorption (as delivered with usual NRT products) is slower and subject to first-pass metabolism. Therefore, contrary to ENDS, usual NRT products may pose less abuse liability about the risk of initiate or maintain nicotine dependence. However, in perfect accordance with the global objective of smoking reduction or cessation, the development of more effective ENDS technologies appear essential. The main challenge consists in offering an increased and well-controlled nicotine delivery to the vapers. Smokers using ENDS for smoking cessation are already nicotine-dependent persons. But it is possible that technological improvement of ENDS in nicotine delivery efficacy could, as a side effect, make vapers more addicted to nicotine. However, the main issue is that smokers have sometimes difficulties to adequately satisfy their nicotine cravings using ENDS technologies currently on the market. Indeed, ENDS differ remarkably from tobacco cigarettes in terms of systemic nicotine delivery. Although nicotine content in the ENDS aerosol is in the same order of magnitude compared to the mainstream of conventional cigarette, clinical data indicate that the nicotine absorption potential is significantly lower for vapers compared to smokers^18^. An improvement in the efficacy of ENDS to deliver aerosol nicotine can be highly beneficial for smokers, with the ability to adjust use patterns for smoking cessation purpose. We expect that new-generation high-power ENDS will deliver well-controlled nicotine at a faster rate and will be more efficient for smokers to satisfy their nicotine need. Consequently, these ENDS technologies could contribute to decrease the number of active smokers and to limit the risk to make vapers more addicted to nicotine. Technological research focusing on airborne nicotine flux into the ENDS aerosol represents an essential step to develop in a next future more effective ENDS technologies, and thus to rise smoking cessation and reduction.

In this frame, two main options can be followed:

(i) To increase the aerosol nicotine concentration. However, in Europe, the newly revised Tobacco Products Directive proposes to regulate ENDS as a tobacco related product^19^. These new rules implemented across the European Union member states applied since May 2016 specified how tobacco related products can be sold, presented and manufactured. Especially, this regulatory decision fixed an upper limit at 20 mg/mL of nicotine concentration in refill liquid. Thus, the increase of nicotine concentration in refill liquid in order to rise the aerosol nicotine concentration becomes impossible. Other ways such as new formulations of refill liquid (*e.g*. playing on the PG/VG ratio, development of new solvents VG-free, etc.) should be explored.
(ii) On the other hand, to improve the nicotine absorption level. Longer and deeper puffs increase the nicotine absorption level. Vapers and clinicians have to be aware of this impact of puffing behaviors. ENDS technical features can also be adjusted (mainly power level which can be variable and easy to change using recent high-power ENDS) to modify both the aerosol output and the airborne particle size distribution. These two parameters are critical for the nicotine delivery to lungs.

## Conclusion

This work examined the nicotine delivery from the refill liquid to the aerosol with similar experimental protocol (cascade impactor set-up) and endpoints (MMAD, dosage of active product for each size-fraction of aerodynamic diameter) that methodologies used for aerosol drug delivery. This paper demonstrated that the particle size distribution of the airborne refill liquid *vs*. aerodynamic diameter is perfectly representative of the particle size distribution of the aerosol nicotine. This result indicates that new generation high-power ENDS associated with PG/VG based solvent (whatever the PG/VG ratio) are very efficient to generate carrier-droplets containing nicotine molecules with a constant concentration (whatever the size-fractions in the submicron range). Furthermore, significant change of the nicotine concentration seems to occur when vaping with high-power ENDS. The proof was made that the aerosol nicotine concentration was lower compared to the initial nicotine concentration of the refill liquid. Under our experimental conditions, results highlighted a disparity between the nicotine content claimed on refill liquid packaging and the aerosol nicotine concentration. The portion of aerosol nicotine delivered clearly indicate that a part of the nicotine may be left in the ENDS upon depletion, and consequently that a portion of the nicotine may not be transferred to the user. These findings provide a better understanding on how to enhance the nicotine delivery from the refill liquid to the aerosol.
